# Effects of COVID-19 Mental Health Interventions Among Children, Adolescents, and Adults Not Quarantined or Undergoing Treatment Due to COVID-19 Infection: A Systematic Review of Randomised Controlled Trials

**DOI:** 10.1177/07067437211070648

**Published:** 2022-03-11

**Authors:** Olivia Bonardi, Yutong Wang, Kexin Li, Xiaowen Jiang, Ankur Krishnan, Chen He, Ying Sun, Yin Wu, Jill T. Boruff, Sarah Markham, Danielle B. Rice, Ian Thombs-Vite, Amina Tasleem, Tiffany Dal Santo, Anneke Yao, Marleine Azar, Branka Agic, Christine Fahim, Michael S. Martin, Sanjeev Sockalingam, Gustavo Turecki, Andrea Benedetti, Brett D. Thombs

**Affiliations:** 1 113635 Lady Davis Institute for Medical Research, Jewish General Hospital, Montreal, Quebec, Canada; 2 Department of Psychiatry, 5620 McGill University, Montreal, Quebec, Canada; 3 Schulich Library of Physical Sciences, Life Sciences, and Engineering, 5620 McGill University, Montreal, Quebec, Canada; 4 Department of Biostatistics and Health Informatics, King's College London, London, United Kingdom; 5 Department of Psychology, 5620 McGill University, Montreal, Quebec, Canada; 6 7978 Centre for Addiction and Mental Health, Toronto, Ontario, Canada; 7 Dalla Lana School of Public Health, 7938 University of Toronto, Toronto, Ontario, Canada; 8 Li Ka Shing Knowledge Institute, Unity Health Toronto, Toronto, Ontario, Canada; 9 School of Epidemiology and Public Health, University of Ottawa, Ontario, Canada; 10 Correctional Service of Canada, Ottawa, Ontario, Canada; 11 149914 Department of Psychiatry, 7938 University of Toronto, Toronto, Ontario, Canada; 12 McGill Group for Suicide Studies, Douglas Mental Health University Institute, 5620 McGill University, Montreal, Quebec, Canada; 13 Department of Epidemiology, Biostatistics, and Occupational Health, 5620 McGill University, Montreal, Quebec, Canada; 14 Department of Medicine, 5620 McGill University, Montreal, Quebec, Canada; 15 Respiratory Epidemiology and Clinical Research Unit, 5620 McGill University Health Centre, Montreal, Quebec, Canada; 16 Department of Educational and Counselling Psychology, 5620 McGill University, Montreal, Quebec, Canada; 17 Biomedical Ethics Unit, 5620 McGill University, Montreal, Quebec, Canada

**Keywords:** coronavirus, COVID-19, mental health interventions, psychological outcomes, living systematic review

## Abstract

**Objectives:**

Our objective was to assess the effects of mental health interventions for children, adolescents, and adults not quarantined or undergoing treatment due to COVID-19 infection.

**Methods:**

We searched 9 databases (2 Chinese-language) from December 31, 2019, to March 22, 2021. We included randomised controlled trials of interventions to address COVID-19 mental health challenges among people not hospitalised or quarantined due to COVID-19 infection. We synthesized results descriptively due to substantial heterogeneity of populations and interventions and risk of bias concerns.

**Results:**

We identified 9 eligible trials, including 3 well-conducted, well-reported trials that tested interventions designed specifically for COVID-19 mental health challenges, plus 6 other trials with high risk of bias and reporting concerns, all of which tested standard interventions (e.g., individual or group therapy, expressive writing, mindfulness recordings) minimally adapted or not specifically adapted for COVID-19. Among the 3 well-conducted and reported trials, 1 (*N*  =  670) found that a self-guided, internet-based cognitive-behavioural intervention targeting dysfunctional COVID-19 worry significantly reduced COVID-19 anxiety (standardized mean difference [SMD] 0.74, 95% confidence interval [CI], 0.58 to 0.90) and depression symptoms (SMD 0.38, 95% CI, 0.22 to 0.55) in Swedish general population participants. A lay-delivered telephone intervention for homebound older adults in the United States (*N*  =  240) and a peer-moderated education and support intervention for people with a rare autoimmune condition from 12 countries (*N*  =  172) significantly improved anxiety (SMD 0.35, 95% CI, 0.09 to 0.60; SMD 0.31, 95% CI, 0.03 to 0.58) and depressive symptoms (SMD 0.31, 95% CI, 0.05 to 0.56; SMD 0.31, 95% CI, 0.07 to 0.55) 6-week post-intervention, but these were not significant immediately post-intervention. No trials in children or adolescents were identified.

**Conclusions:**

Interventions that adapt evidence-based strategies for feasible delivery may be effective to address mental health in COVID-19. More well-conducted trials, including for children and adolescents, are needed.

## Abrégé

**Objectifs:**

Notre objectif était d’évaluer les effets des interventions de santé mentale pour les enfants, les adolescents, et les adultes qui ne sont pas en quarantaine ni ne subissent un traitement en raison d’une infection de la COVID-19.

**Méthodes:**

Nous avons cherché dans 9 bases de données (2 en chinois) du 31 décembre 2019 au 22 mars 2021. Nous avons inclus des essais randomisés contrôlés des interventions pour aborder les problèmes de santé mentale de la COVID-19 chez les personnes non hospitalisées ni en quarantaine en raison d’une infection de la COVID-19. Nous avons fait un résumé descriptif des résultats à cause de l’hétérogénéité substantielle des populations et des interventions, et des préoccupations quant au risque d’un biais.

**Résultats:**

Nous avons identifié 9 essais admissibles, notamment 3 essais bien menés et bien rapportés qui vérifiaient les interventions conçues spécifiquement pour les problèmes de santé mentale de la COVID-19, plus 6 autres essais comportant un risque de biais élevé et des préoccupations de rapport, et tous vérifiaient les interventions normales (p. ex., la thérapie individuelle ou de groupe, l’écriture expressive, les enregistrements de pleine conscience) minimalement adaptées ou non spécifiquement adaptées à la COVID-19. Parmi les 3 essais bien menés et bien rapportés, l’un (N  =  670) a constaté qu’une intervention autoguidée, sur Internet, cognitivo-comportementale qui ciblait une possibilité dysfonctionnelle de la COVID-19 réduisait significativement l’anxiété de la COVID-19 (différence moyenne normalisée [DMN] 0,74, IC à 95% 0,58 à 0,90) et les symptômes de dépression (DMN 0,38, IC à 95% 0,22 à 0,55) chez les participants de la population générale suédoise. Une intervention téléphonique par des non-professionnels à des adultes âgé à la maison aux États-Unis (N  =  240) et une intervention éducative de soutien modérée par les pairs pour les personnes ayant une rare condition auto-immune de 12 pays (N  =  172) ont significativement amélioré l’anxiété (DMN 0,35, IC à 95% 0,09 à 0,60; DMN 0,31, IC à 95% 0,03 à 0,58) et les symptômes dépressifs (DMN 0,31, IC à 95% 0,05 à 0,56; DMN 0,31, IC à 95% 0,07 à 0,55) 6 semaines après l’intervention, mais celles-ci n’étaient pas significatives immédiatement après l’intervention. Aucun essai sur les enfants ou les adolescents n’a été identifié.

**Conclusions:**

Les interventions qui adaptent les stratégies fondées sur des données probantes pour une prestation faisable peuvent être efficaces pour aborder la santé mentale dans la COVID-19. Il faut plus d’essais bien menés, notamment pour les enfants et les adolescents.

## Introduction

The SARS-CoV-2 coronavirus disease (COVID-19) pandemic has caused over 3 million deaths worldwide and disrupted social, educational, and economic activities.^1,2^ Internationally, people have faced long periods of lockdown and isolation. There are concerns about effects on mental health,^2-4^ particularly among groups vulnerable to health or social and economic effects of COVID-19, including older individuals; children and adolescents; people with pre-existing medical or mental health conditions; essential services personnel; and individuals marginalized due to poverty, race/ethnicity, or other factors.^5^ Vaccination is underway, but lockdown restrictions will likely continue, at least intermittently, and mental health implications may persist.^2^

COVID-19 mental health challenges may include loneliness, boredom, grief and loss, depression, stress, worry, fear, burnout, and anxiety.^2-8^ Scalable mental health interventions, which are interventions that can be feasibly delivered to large numbers of people affected by adversity, are needed.^9^ These could include non-specialist-delivered or self-help versions of evidence-based interventions, guided group-based interventions, peer-support interventions, or interventions delivered via the internet rather than in person, for example.^9,10^

We identified 5 systematic reviews^11-15^ that have attempted to synthesize evidence on mental health interventions for non-hospitalised children, adolescents, or adults in COVID-19, but none included any randomised controlled trials (RCTs) from COVID-19 for people not quarantined or undergoing treatment due to COVID-19. End dates of searches were between April and September 2020, and none are ongoing.

Living systematic reviews^16^ are systematic reviews that are continually updated to provide up to date evidence. They are logistically challenging but highly valuable when (1) important decisions to be made merit the resources involved; (2) low certainty in existing evidence poses a barrier to decision-making; and (3) new emerging evidence may inform decisions.^16^ Timely evidence is needed to support mental health responses to COVID-19.

We are conducting living systematic reviews^3,4^ of changes in mental health symptoms during COVID-19 and effects of mental health interventions, both for people with COVID-19 infection or exposure and for community-based public mental health. The objective of the present report is to synthesize evidence from RCTs on mental health interventions for community-based children, adolescents, and adults not quarantined or undergoing treatment due to COVID-19 infection.^3,4^

## Methods

Our systematic review was registered in the PROSPERO prospective register of systematic reviews (CRD 42020179703), and a protocol was uploaded to the Open Science Framework (https://osf.io/96csg/) prior to initiation. Results are reported in accordance with the PRISMA statement.^17^ Results are also posted online (https://www.depressd.ca/research-question-3-intervention). The present report includes RCTs of interventions conducted with people not quarantined or undergoing treatment due to COVID-19 infection, which is a subset of trials included in our main systematic review of interventions.

### Eligible Studies

Our main living systematic review of interventions is collecting results from randomised or non-randomised trials of mental health interventions conducted in any population during COVID-19. For trials to be included among those with results posted online in the main review, all participants had to be enrolled after December 31, 2019, when China first reported on COVID-19 to the World Health Organization.^18^ Eligible interventions included any intervention described as designed to address COVID-19 mental health challenges or primarily addressing mental health symptoms from COVID-19. Trials that were not mental health interventions and primarily targeted non-mental health outcomes (e.g., exercise with primary outcome physical activity) were excluded, even if mental health outcomes were reported. Eligible comparators included: (1) inactive control conditions (e.g., no treatment, waitlist) and (2) other eligible interventions. Eligible outcomes were defined broadly and included general mental health, mental health quality of life, anxiety symptoms, depression symptoms, stress, loneliness, anger, grief, burnout, and other emotional states. To be eligible, trials had to report outcomes collected at least 1 week after intervention initiation and include at least 10 total participants. There were no restrictions on language or publication format.

The present report focuses on a subset of psychological interventions from the main review that were designed to address COVID-19 mental health among people not known to have COVID-19. Thus, it does not include trials done exclusively with hospitalised patients or persons quarantined due to COVID-19 infections or exposure, because they face different challenges than people not infected with COVID-19. Additionally, to include only trials likely to provide evidence useful for policy and practice decisions, we excluded trials of brief single-session interventions (e.g., 30 min) with no subsequent follow-up, and we excluded non-randomised studies. We did not include non-randomised studies because such studies are highly prone to bias when intervention and control groups are self-selected or there is no control group. Results from pre-post analyses of non-randomised studies without a control group are not possible to interpret unless there is precise knowledge of the natural trajectory of symptoms or if one can safely assume that symptoms will not change over time without intervention. Even in normal times, however, this is not the case for mental health trials. Participants often seek mental health services and enrol in trials when they are experiencing high levels of symptoms, and regression to the mean is common.^19-23^ Approximately 40% of participants assigned to placebo groups in drug trials or no-treatment groups in psychological intervention trials for major depression, for instance, achieve remission.^24^ The Cochrane Collaboration discourages the inclusion of evidence from non-randomised studies when conducting trials is feasible and when evidence from non-randomised trials is subject to these kinds of biases.^25^ Non-included trials from the main systematic review, including non-randomised studies, are shown in supplemental material.

Additionally, the high volume of poor-quality research being published on COVID-19 is a barrier to synthesis,^26^ and we encountered many trials that were of extremely poor quality, of unclear origin and sponsorship, and reported effect sizes that, in some cases, exceeded plausibility. Thus, we contacted the authors of all included studies by email up to 2 times and requested that they verify the authenticity of published methods and results and confirm the accuracy of our extracted data. Authors of studies published in Chinese-language journals were contacted with text in both English and Chinese. We did not include unverified trials in our main report but instead show results in supplemental material.

### Identification and Selection of Eligible Studies

The same search strategies were used for all research questions in our systematic reviews. We searched MEDLINE (Ovid), PsycINFO (Ovid), CINAHL (EBSCO), EMBASE (Ovid), Web of Science Core Collection: Citation Indexes, China National Knowledge Infrastructure, Wanfang, medRxiv (preprints), and Open Science Framework Preprints (preprint server aggregator) using a strategy designed and built by an experienced health sciences librarian. The China National Knowledge Infrastructure and Wanfang databases were searched using Chinese search terms based on the English-language search strategy. The rapid project launch did not allow for formal peer review, but COVID-19 terms were developed in collaboration with other librarians working on the topic. See Supplement 1. Our initial search was conducted from December 31, 2019, to April 13, 2020, then automated searches were set for daily updates. On December 28, 2020, we converted to weekly updates to improve processing efficiency.

Search results were downloaded into the systematic review software DistillerSR (Evidence Partners, Ottawa, Canada), where duplicate references were identified and removed. Two independent reviewers evaluated titles and abstracts in random order. If either reviewer deemed a study potentially eligible, a full-text review was completed, also by 2 independent reviewers. Discrepancies at the full-text level were resolved through consensus, with a third investigator consulted as necessary. To ensure the accurate identification of eligible studies, a coding guide with inclusion and exclusion criteria was developed and pretested, and all team members were trained over several sessions. See Supplement 2.

### Data Extraction and Synthesis

For each included study, 1 reviewer extracted data using a pre-specified standardized form, and a second reviewer validated extracted data. Reviewers extracted (1) publication characteristics (e.g., first author, journal); (2) population characteristics (e.g., country, eligibility criteria, recruitment method, number of participants, age, sex, or gender); (3) COVID-19 characteristics (e.g., time during pandemic); (4) intervention and control group characteristics, including elements important for the scalability of interventions (delivery format; individual, group, or self-administration; personnel required); (5) mental health outcomes; (6) risk of bias; and (7) adequacy of intervention reporting. If sufficient information to calculate effect sizes for 1 or more outcomes was not provided, we contacted authors to obtain missing information.

We used the 2011 version of the Cochrane Collaboration risk of bias tool.^27^ The tool has 7 domains, including random sequence generation, allocation concealment, blinding of participants and personnel, blinding of outcome assessment, incomplete outcome data, selective outcome reporting, and other sources of bias. Studies were rated low, unclear, or high risk on each domain.

We used the Template for Intervention Description and Replication (TIDieR) checklist to evaluate the degree that interventions were reported adequately for replication in research or practice.^28^ The checklist is comprised of 12 items that assess reporting of intervention name; rationale or theory underlying the intervention; physical or informational material used; procedures and processes; provider and background; delivery mode (e.g., group, face-to-face); location where delivered and necessary infrastructure; number of sessions, schedule, and duration; if tailoring was done and how; any modifications made; if adherence or fidelity was assessed and how; and, if assessed, the extent to which the intervention was delivered as planned.

For included trials, if not provided, we calculated between-groups standardized mean differences (SMDs) using Hedges’ g with 95% confidence intervals (CIs).^29^ We did not pool results across trials because of substantial heterogeneity of populations, interventions, and outcomes and concerns about the risk of bias. Instead, we reported results descriptively.

### Protocol Amendments

Our systematic review was quickly designed and initiated in April 2020. Several amendments or clarifications were made subsequently. First, we changed from daily to weekly search updates on December 28, 2020, for more efficient reference processing. Second, on January 27, 2021, we made a minor change to search strategies to incorporate a new physical distancing subject heading created for COVID-19. Third, we made several amendments to Chinese-language search strategies to facilitate processing (see Supplement 1). Fourth, we added the TIDieR^28^ checklist to assess intervention reporting quality. Fifth, we clarified that we only included trials that initiated participant enrolment after December 31, 2019. Sixth, we clarified criteria for assessing whether an intervention addressed mental health related to COVID-19; see Supplement 2. Seventh, we decided to separately report trials of interventions done with people not infected with COVID-19 or quarantined due to exposure, due to major differences in challenges faced by these groups and intervention approaches compared to those with COVID-19. Eighth, because we have encountered many trial reports of poor quality with seemingly implausible results, this report only includes trials whose authors verified the accuracy of their report and our extracted data; results of other trials are in supplemental material.

## Results

### Search Results and Selection of Eligible Studies

As of March 22, 2021, our searches identified 45,777 unique citations. Of these, 45,536 were excluded after title and abstract review and 146 after full-text review, leaving 95 trials, of which 59 evaluated interventions for people hospitalised or quarantined due to COVID-19, 10 assessed single-session interventions without subsequent follow-up, four were non-randomised trials, and 13 were not verified by authors (6 without author contact information in publication or online; 7 no response), leaving 9 eligible, verified RCTs for inclusion.^30-38^ See PRISMA flow diagram in Supplement 3.

### Characteristics of Included Trials

Table 1 shows the characteristics of included RCTs. See Supplement 4 for characteristics (plus outcomes, risk of bias, intervention reporting) of otherwise eligible but unverified trials and Supplement 5 for trials with hospitalised or quarantined individuals, trials of brief interventions without follow-up, and non-randomised trials. Of the 9 included trials, 3 trials^30-32^ tested interventions designed specifically to address mental health challenges in COVID-19, and 6^33-38^ tested standard interventions that were only minimally adapted or not adapted for COVID-19.

**Table 1.** Characteristics of Included Trials.

Author Dates Country(ies) Registration	Participants	Intervention Comparator	COVID-19-specific and Scalability Aspects: Delivery Format Individual/Group/Self-admin Professional/Lay/No personnel	N Analyzed: Intervention/Comparator	Outcome Time to Follow-up Post-Randomization and Domain(s)^a^	Mean (*SD*) Age	% Female or Women
Kahlon et al.^30^ 07/2020 to 09/2020 USA NCT04595708 (retrospective)	Homebound older adults receiving services through a Meals on Wheels organization	Volunteers trained in empathetic conversational techniques called participants over 4 weeks, daily for the first 5 days then 2–5 calls per week. Calls were targeted to be less than 10 min; however, callers reported that calls could run longer No calls	Designed to address loneliness in homebound meal recipients isolated due to COVID-19 Telephone Individual Lay volunteer delivery	120/120	4 weeks Anxiety; Depression; Mental Health Function; Loneliness	69 (12)	79%
Thombs et al.^31^ 04/2020 to 07/2020 Canada, USA, France, UK, Australia, 7 others NCT04335279	Adults with systemic sclerosis and at least mild anxiety (PROMIS Anxiety 4a v1.0 ≥ 55) recruited from a multinational cohort	4-week, 3× per week, 90-min videoconference group sessions focusing on leisure activities, mental health coping, and social support Waitlist	Designed with patients to target COVID-19 anxiety through evidence-based strategies and social support Videoconference Group Mixed professional and peer volunteer delivery	86/86	4 weeks and 10 weeks Anxiety; Depression; Loneliness; Fear	55 (11)	94%
Wahlund et al.^32^ 05/2020 to 07/2020 Sweden NCT04341922	Swedish adults with difficulty controlling worry about COVID-19, excluding those with moderate to severe depression or suicide risk, recruited via media from general population	3 weeks of self-directed, established online cognitive behavioural intervention for worry-related problems plus additional modules adapted specifically for dysfunctional COVID-19 worry Waitlist	Evidence-based cognitive behavioural strategies to address worry adapted for dysfunctional COVID-19 worry Internet Self-administered No personnel to deliver	335/335	3 weeks COVID-19 Anxiety; Depression	46 (14)	82%
Al-Alawi et al.^33^ 04/2020 to 07/2020 Oman NCT04378257	Adults aged 18–65 from Oman with PHQ-9 ≥ 12 or GAD-7 ≥ 10 and no pre-existing mental health or substance use disorders or suicide ideation, recruited from a list of online survey respondents	6 weekly videoconference-based individual therapy sessions based on principles of cognitive behavioural therapy and acceptance and commitment therapy Minimal: weekly newsletter with self-help tips	No COVID-19 adaptations reported Videoconference Individual Professional	22/24	6 weeks Anxiety; Depression	29 (9)	78%
Pheh et al.^34^ NR Malaysia Not registered	Adults recruited from social media	Single ultra-brief online mindfulness-based journaling exercise Single ultra-brief online journaling exercise not based on mindfulness	Standard mindfulness journaling minimally adapted to reflect on movement restrictions Internet Self-administered No personnel to deliver	33/28^b^	3 weeks Anxiety; Mental Health Function; Fear	NR	NR
Pizarro-Ruiz et al.^35^ 04/2020 to 05/2020 Spain Not registered	Students in social education or nursing from a single university, recruited via email	Daily app-based 15-min mindfulness sessions for 2 weeks using publicly available app Daily app-based 15-min mind training (e.g., attention, memory) sessions for 2 weeks using publicly available app	No COVID-19 adaptations reported Internet Self-administered No personnel to deliver	89/75	2 weeks Mental Health Function	22 (6)	83%
Shabahang^36^ NR Iran Not registered	Students from a single university with significant coronavirus anxiety who were not receiving active psychological treatments; recruitment method not provided	Group-based 90-min cognitive behavioural therapy sessions focused on health anxiety delivered 5 days per week for 2 weeks Waitlist	Included lecture by virologist on COVID-19 but no other COVID-19 adaptations reported Not reported Group Professional	75/75	2 weeks Anxiety; Depression	NR	NR
Vukčević Marković et al.^37^ NR Serbia ISRCTN17898730 (retrospective)	Serbian adults recruited via social media	5 online 20-min expressive writing sessions over 2 weeks (3 days between sessions), during which participants were instructed to write anything that came to mind regarding COVID-19 No intervention	Minimal adaptation of expressive writing by using COVID-19 theme Internet Self-administered No personnel to deliver	2 weeks 48/56 4 weeks 36/38	2 weeks and 6 weeks Anxiety; Depression; Mental Health Function; Stress	32 (10)	74%
Yang et al.^38^ NR China Not registered	Chinese students from a single university at home due to COVID lockdown; recruitment method not provided	Audio-recorded 30-min mindfulness-based stress reduction session once every 2 days for 10 days No intervention	Minimal adaptation by including mindfulness exercise on accepting COVID-19-related negative thoughts and affect Internet Self-administered No personnel to deliver	53/51	2 weeks Anxiety; Depression; Mental Health Function; Stress	19 (1)	53%

GAD-7  =  Generalized Anxiety Disorder scale; NR  =  not reported; PHQ-9  =  Patient Health Questionnaire-9.

^a^ Specific scales used in each trial are shown in Table 3.

^b^ Only follow-up data (*N*  =  61), but not results from assessment immediately following the single-session intervention, were eligible for inclusion and are reported here.

The 3 trials^30-32^ of interventions designed specifically to address mental health challenges in COVID-19 all included aspects to promote scalability and access; all were compared to no intervention or waitlist control groups. Two^30,31^ used lay-delivered or peer-support interventions with groups of vulnerable individuals. The third^32^ used an online cognitive behavioural therapy intervention to address COVID-19 worry in the general population.

A trial from the United States (*N*  =  240)^30^ tested the effects of 4 weeks of layperson-delivered telephone calls to racially and ethnically diverse homebound older adults receiving home meal services through a Meals on Wheels organization (mean [*SD*] age  =  69 [12], 79% women, 100% chronic medical condition) on anxiety, depressive symptoms, general mental health function, and loneliness. The investigators trained university students in empathetic conversational skills (e.g., prioritizing listening, eliciting conversation on topics of interest to participants), and each caller supported 6 to 9 participants. Calls, which were targeted to be <10 min, were done on 5 days in the first week and 2 to 5 days in the following 3 weeks.

A second trial^31^ (*N*  =  172) randomised people with the rare autoimmune disease systemic sclerosis, or scleroderma, from 12 countries to receive 4 weeks (3 times per week) of a multifaceted group videoconference-based intervention or waitlist control. It tested the effects of the intervention, which combined activity engagement, education and practice in mental health coping strategies, and peer support on outcomes that included anxiety, depressive symptoms, fear, and loneliness. Groups included 6 to 10 participants and were moderated by peers previously trained as support group leaders.

The third trial (*N*  =  670)^32^ tested effects on COVID-19-related anxiety and depressive symptoms after receiving 3 weeks of access to a self-guided online cognitive behavioural intervention. Adults in the Swedish general population were recruited through advertising on national television, newspapers, and social media and randomised to the intervention or waitlist control. The intervention was based on established cognitive behavioural intervention principles adapted to specifically address dysfunctional COVID-19 worry. The project was done in collaboration with public health authorities and made available to the public free of charge following testing.

The 6 other trials^33-38^ tested standard interventions minimally adapted or not specifically adapted for mental health during COVID-19 and were conducted in Oman (*n* =  46),^33^ Malaysia (*n*  =  61),^34^ Spain (*n*  =  164),^35^ Iran (*n* =  150),^36^ Serbia (*n*  =  104),^37^ and China (*n*  =  104).^38^ Participants were recruited via social media,^34,37^ an internet survey,^33^ and a university email list,^35^ in 2 trials, recruitment method was not reported.^36,38^ Two trials tested standard cognitive behavioural therapy delivered individually (6 sessions; comparator  =  self-help newsletter)^33^ or in groups (10 sessions; comparator  =  waitlist)^36^; both reported targeting mental health symptoms from COVID-19, but neither described COVID-19-specific intervention adaptations apart from providing information on COVID-19.^36^ Two interventions tested standard self-guided mindfulness journaling (1 session; comparator  =  journaling not based on mindfulness)^34^ or expressive writing (5 sessions; comparator  =  no intervention)^37^ adapted by instructing participants to write about experiences during the pandemic. Two tested self-guided mindfulness apps (14 sessions; comparator  =  cognitive training app)^35^ or audio recordings (5 sessions; comparator  =  no intervention)^38^ that were described as targeting COVID-19 mental health symptoms but did not describe adaptations for COVID-19 challenges.

### Risk of Bias and Adequacy of Intervention Description

Risk of bias assessments is shown in Table 2 and the adequacy of intervention descriptions in Supplement 6. For all 3 trials that tested interventions designed specifically to be delivered feasibly and address COVID-19 mental health concerns,^30-33^ the risk of bias was low for random sequence generation, allocation concealment, incomplete outcome data, and other bias sources. It was high for all 3 trials for blinding of participants and personnel and outcome assessment, since outcomes involved symptom self-report by non-blinded participants. Two trials^31,32^ were rated low for selective outcome reporting because outcomes matched a priori registered outcomes; the other trial^30^ was rated unclear because registration was retrospective. Interventions were well-described for all 3 trials with zero,^31^ one,^32^ and three^30^ of 12 items rated no or partial on the TIDieR Checklist.

**Table 2.** Risk of Bias of Included Trials.

Author	Random sequence generation	Allocation concealment	Blinding of participants/personnel	Blinding of outcome assessment	Incomplete outcome data		Selective reporting	Other bias
Kahlon et al^30^	Low	Low	High^a^	High^a^	Low		Unclear^b^	Low
Thombs et al.^31^	Low	Low	High^a^	High^a^	Low		Low	Low
Wahlund et al.^32^	Low	Low	High^a^	High^a^	Low		Low	Low
Al-Alawi et al.^33^	Low	Low	High^a^	High^a^	High^c^		Low	Low
Pheh et al.^34^	Unclear^d^	Unclear^e^	Low^f^	Low^f^	High^g^		Unclear^h^	Low
Pizarro-Ruiz et al.^35^	Unclear^d^	Unclear^e^	Low^f^	Low^f^	High^i^		Unclear^h^	High^j^
Shabahang^36^	Unclear^d^	Unclear^e^	High^a^	High^a^	High^k^		Unclear^h^	Low
Vukčević Marković et al.^37^	Low	Unclear^e^	High^a^	High^a^	Unclear^l^ High^l^		Unclear^b^	High^m^
Yang et al.^38^	Unclear^d^	Unclear^e^	High^a^	High^a^	Low		Unclear^h^	Low

^a^ Participants (and in some cases study personnel) were not blinded, and outcomes were assessed via participant self-report.

^b^ Registered retrospectively.

^c^ Small number of participants in each arm and loss to follow-up of 26% and 20%.

^d^ The randomisation procedure was not described.

^e^ Method of allocation concealment not described.

^f^ Randomised to 1 of 2 online apps and most likely blind to study objectives.

^g^ Only 30% of randomised included in analyses.

^h^ No pretrial registration or publicly accessible protocol.

^i^ Excluded all participants who missed intervention sessions or did not complete all assessments but did not provide numbers.

^j^ Baseline differences in outcome measures between groups large (max Hedges’ g  =  0.57).

^k^ Excluded participants who missed intervention sessions or deemed uncooperative but did not provide numbers.

^l^ Loss to follow-up 13% at first assessment but N  =  12 in intervention and N  =  4 in control; loss to follow-up 38% at second assessment.

^m^ Large discrepancy in women randomised to intervention (92% of 89) and control (72% of 75) and other imbalances raise concern about randomisation.

Among the 6 other trials,^33-38^ 1 trial^33^ had 3 high-risk ratings, and the other 5 trials^34-38^ had between four and six unclear or high ratings out of 7 risk of bias items. Most interventions were described sub-adequately; all had 3 to 6 no or partial TIDieR Checklist ratings. Interventions either did not evaluate intervention delivery fidelity or adherence or were rated as no or partial reporting if evaluation did take place.

### Mental Health Outcomes

**Figure 1.**
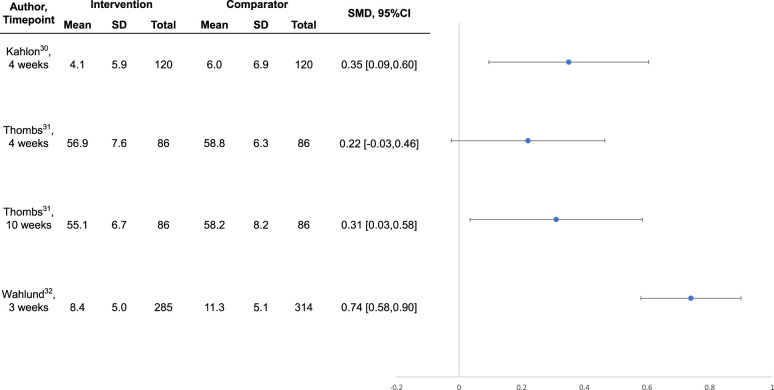
Forest plot of effects on symptoms of anxiety among well-conducted and reported interventions designed to address COVID-19 mental health challenges.

**Figure 2.**
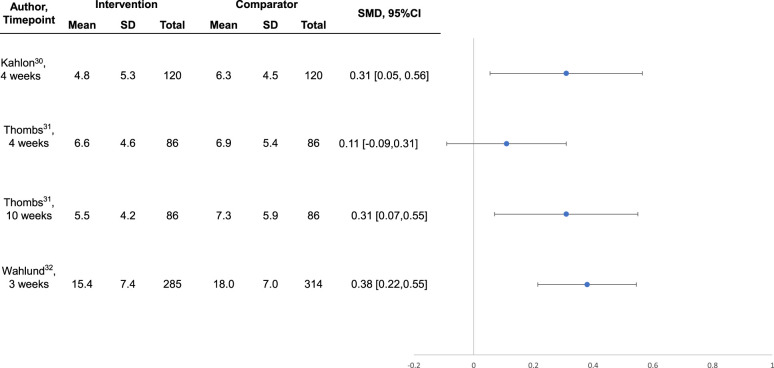
Forest plot of effects on symptoms of depression among well-conducted and reported interventions designed to address COVID-19 mental health challenges.

Intervention effects are shown in Table 3. Compared to no intervention or waitlist control, the 3 interventions^30-32^ that were well-conducted and reported, all of which were designed specifically for COVID-19, reduced general or COVID-19-specific anxiety symptoms between SMD  =  0.31 (95% CI, 0.03 to 0.58)^23^ and 0.74 (95% CI, 0.58 to 0.90)^32^ at the last trial assessments. Symptoms of depression were reduced by SMD  =  0.31 (95% CI, 0.05 to 0.56)^22^ to 0.38 (95% CI, 0.22 to 0.55).^25^ For the trial in systemic sclerosis,^31^ although effects were statistically significant 10-week post-randomisation, they were not statistically significant immediately following the 4-week intervention (see Figures 1 and 2).

**Table 3.** Standardized Mean Difference (SMD) Effect Sizes of Mental Health Outcomes^a^.

Author Dates Country	Anxiety SMD (95% CI)		Depression SMD (95% CI)		Mental Health Function SMD (95% CI)		Loneliness SMD (95% CI)		Fear SMD (95% CI)		Stress SMD (95% CI)	
Kahlon et al.^30^ 07/2020 to 09/2020 USA	GAD-7	4 weeks 0.35 (0.09, 0.60)	PHQ-8	4 weeks 0.31 (0.05, 0.56)	SF-12 MCS	4 weeks 0.46 (0.20, 0.72)	ULS-3 De Jong	4 weeks 0.48 (0.22, 0.74) 4 weeks 0.17 (−0.08, 0.42)	———-	———-	———-	———-
Thombs et al.^31^ 04/2020 to 07/2020 Canada, USA, France, UK, Australia, 7 others	PROMIS Anxiety 4a v1.0	4 weeks 0.22 (−0.03, 0.46) 10 weeks 0.31 (0.03, 0.58)	PHQ-8	4 weeks 0.11 (−0.09, 0.31) 10 weeks 0.31 (0.07, 0.55)	———-	———-	ULS-6	4 weeks 0.09 (−0.12, 0.31) 10 weeks 0.02 (−0.22, 0.26)	CFQCMC	4 weeks 0.12 (−0.06, 0.29) 10 weeks −0.03 (−0.22, 0.16)	———-	———-
Wahlund et al.^32^ 05/2020 to 07/2020 Sweden	GAD-7 (COVID)^b^	3 weeks 0.74 (0.58, 0.90)	MADRS	3 weeks 0.38 (0.22, 0.55)	———-	———-	———-	———-	———-	———-	———-	———-
Al-Alawi et al.^33^ NR Oman	GAD-7	6 weeks 0.78 (0.17,1.38)	PHQ-9	6 weeks 0.82 (0.21,1.43)	———-	———-	———-	———-	———-	———-	———-	———-
Pheh et al.^34^ NR Malaysia	GAD-7	2 weeks −0.05 (−0.56, 0.45)	———-	———-	SUD WHO-5	2 weeks 0.11 (−0.40, 0.61) 2 weeks −0.20 (−0.70, 0.31)	———-	———-	FCS	2 weeks 0.20 (−0.31, 0.70)	———-	———-
Pizarro-Ruiz et al.^35^ 04/2020 to 05/2020 Spain	———-	———-	———-	———-	PANAS-PA PANAS-NA SWLS	2 weeks 0.41 (0.10, 0.72) 2 weeks 0.46 (0.15, 0.78) 2 weeks 0.48 (0.22, 0.74)	———-	———-	———-	———-	———-	———-
Shabahang^36^ NR Iran	SHAI	2 weeks 1.14 (0.80, 1.49)	BDI-II	2 weeks 1.11 (0.76, 1.45)	———-	———-	———-	———-	———-	———-	———-	———-
Vukčević Marković et al.^37^ NR Serbia	DASS (Anx)	2 weeks −0.37 (−0.76, 0.02) 6 weeks −0.32 (−0.78, 0.15)	DASS (Dep)	2 weeks −0.45 (−0.88, −0.06) 6 weeks −0.07 (−0.52, 0.39)	DASS (Total) WHO-5 MANSA	2 weeks −0.66 (−1.06, −0.26) 6 weeks −0.21 (−0.67, 0.25) 2 weeks −0.15 (−0.54,0.24) 6 weeks 0.11 (−0.35, 0.57) 2 weeks −0.13 (−0.51,0.26) 6 weeks 0.23 (−0.43, 0.49)	———-	———-	———-	———-	DASS (Stress)	2 weeks −0.83 (−1.23, −0.42) 6 weeks −0.15 (−0.61, 0.31)
Yang et al.^38^ 02/2020 to 02/2020 China	DASS (Anx)	2 weeks 0.48 (0.09, 0.87)	DASS (Dep)	2 weeks 0.49 (0.10, 0.89)	POMS	2 weeks 0.45 (0.05, 0.84)	———-	———-	———-	———-	DASS (Stress)	2 weeks 0.51 (0.12, 0.90)

BDI-II  =  Beck Depression Inventory-II; CFQCMC  =  COVID-19 Fears Questionnaire for Chronic Medical Conditions; CI  =  confidence interval; DASS  =  Depression Anxiety Stress Scale; De Jong  =  De Jong Giervald Loneliness Scale; FCS  =  Fear of COVID-19 Scale; GAD-7  =  Generalized Anxiety Disorder scale; NR  =  not reported; MANSA  =  Manchester Short Assessment of Quality of Life; PANAS-NA  =  Positive and Negative Affect Scale – Negative Affect; PANAS-PA  =  Positive and Negative Affect Scale – Positive Affect; PHQ-8  =  Patient Health Questionnaire-8; PHQ-9  =  Patient Health Questionnaire-9; POMS  =  Profile of Mood States; SF-12 MCS  =  Short Form 12 Mental Composite Scale; SHAI  =  Short Health Anxiety Inventory; SMD  =  standardized mean difference; SUD  =  subjective units of distress; SWLS  =  Satisfaction with Life Scale; ULS-3  =  UCLA Loneliness Scale-3; ULS-6  =  UCLA Loneliness Scale-6; WHO-5  =  World Health Organization-5 Well-Being Index.

^a^ Outcomes are reported with positive signs favouring the intervention group. Effect sizes reported as provided in publications, if available, prioritising intent-to-treat analyses; if not provided, calculated using Hedges’ g.

^b^ Standard GAD-7 items were reworded to address anxiety and worry about COVID-19 rather than generalized anxiety.

Loneliness was reduced in the trial of lay-delivered phone calls based on 1 measure (SMD  =  0.48, 95% CI, 0.22 to 0.74) but not a second measure (SMD 0.17, 95% CI, −0.08 to 0.42)^30^; neither loneliness nor fear was reduced at either assessment point in the group-based systemic sclerosis intervention.^31^ Two trials tracked adverse effects, and both reported no serious adverse effects.^31,32^

For the 6 trials with a high risk of bias and reporting concerns,^33-38^ reported effects on symptoms of anxiety and depression were between SMD  =  0.78 (95% CI, 0.17 to 1.38) and 1.14 (95% CI, 0.80 to 1.49) for the individual and group cognitive behavioural therapy interventions compared to minimal or no intervention.^33,36^ Effects for single-session mindfulness-based journaling were close to null and not statistically significant compared to a cognitive training app.^34^ For 5 sessions of expressive writing compared to no intervention, of 12 outcome assessments, none favoured the intervention, but 3 were statistically significant and large in favour of the no-intervention control.^37^ The 2 studies that tested app-based^35^ or audio-recorded mindfulness interventions^38^ compared to a cognitive training app or no intervention reported statistically significant effects on several variables in favour of the intervention.^35,38^ The only trial that tracked adverse effects reported no adverse effects.^32^

## Discussion

Scalable, feasibly delivered interventions are needed to address community mental health implications of COVID-19 that will likely persist beyond the pandemic. We identified 3 well-conducted trials, and all tested potentially scalable interventions designed to address COVID-19 mental health in the general public^32^ and among people vulnerable in COVID-19 due to age and pre-existing medical conditions.^30,31^ A self-guided online intervention that targeted COVID-19-specific dysfunctional worry reduced COVID-19 anxiety by SMD  =  0.74 (95% CI, 0.58 to 0.90) and depression symptoms by SMD  =  0.38 (95% CI, 0.22 to 0.55) in the Swedish general public.^32^ A lay-delivered supportive telephone intervention reduced anxiety and depression symptoms and improved mental health function by SMD  =  0.31 (95% CI, 0.05 to 0.56) to SMD  =  0.46 (95% CI, 0.20 to 0.72) among homebound older adults in the United States.^30^ A multifaceted group-based intervention for people with systemic sclerosis from 12 countries, which included peer-led support plus professionally delivered mental health coping strategies, did not significantly reduce mental health outcomes immediately post-intervention, but anxiety (SMD  =  0.31; 95% CI, 0.03 to 0.58) and depression (SMD  =  0.31; 95% CI, 0.07 to 0.55) symptoms significantly improved 6 weeks later.^31^ These effect sizes are comparable to effects from treating the major depressive disorder with antidepressants (SMD  =  0.31)^39^ or from cognitive behavioural therapy for depression in primary care (SMD  =  0.22);^40^ both considered standard health care. The 3 well-conducted trials that we reviewed, similar to most trials of behavioural interventions, were not blinded. Thus, effects may include some degree of bias compared to, for instance, placebo-blinded antidepressant trials. We did not identify any trials of interventions for children or adolescents.

We identified 6 trials that were rated as high risk of bias and were not well-reported; they all tested delivery of standard psychological interventions without significant adaptation for COVID-19, including individual or group-based cognitive behavioural therapy,^33,36^ expressive writing,^34,37^ and self-guided mindfulness apps or audio recordings.^35,38^ The serious concerns, however, about the risk of bias and adequacy of reporting in all of these trials, reduced confidence in results.

Governments and health care providers around the world need effective, scalable interventions to meet the challenges of population mental health in COVID-19, including digital^41^ and other types of interventions.^9,10^ Our findings show that digital interventions for the general public and lay-delivered or peer-supported telephone or videoconference interventions for people who are vulnerable due to age or pre-existing medical conditions may be effective solutions.

The finding that a self-guided internet intervention reduced both anxiety and depression symptoms is consistent with a growing body of evidence that internet-based psychological interventions may be an effective first-line strategy for many people. They are likely not as effective as in-person or guided internet-based therapies and may not be appropriate for people with severe or unremitting illness.^42^ However, consistent with the findings of the study by Wahlund et al.^32^ in the present review, some studies have found that estimates of effectiveness approach those of guided formats, including for anxiety and depressive disorders.^42,43^

Evidence is mixed on the effectiveness of “befriending” or social support-based interventions delivered via video-based communication, online discussion groups and forums, or telephone.^36^ However, factors that appear to be associated with a greater likelihood of effectiveness include shared experiences or characteristics among participants and the ability of participants to speak freely and develop relationships.^44^ These were key components of the 2 trials that used an empathetic telephone calling strategy^30^ and peer-moderated videoconference-based groups for people with the rare autoimmune disease systemic sclerosis.^31^ Although the peer-moderated intervention was done in a rare disease context, none of the intervention strategies were disease-specific, and the approach could be easily adapted for other groups of people with chronic medical conditions.

The evidence from the 3 well-conducted trials included in our review, combined with existing evidence,^36,42-44^ suggest that both self-administered internet approaches and peer support interventions should be considered to address mild mental health concerns in the context of COVID-19 and subsequently. Although there are examples of recent increases in investment to expand access to mental health services, there continue to be substantial shortages in capacity and barriers to access in Canada^45^ and elsewhere.^46^ Scalable interventions, similar to those that have been used successfully in COVID-19 may represent an opportunity to expand support and would be consistent with existing recommendations, such as from the United Kingdom's National Institute for Health and Care Excellence, for stepped care with initial low-intensity approaches for new, mild cases.^47^

We did not identify any trials of interventions for children or adolescents, and it is not known to what degree self-guided or lay- and peer-support interventions would be effective. Unfortunately, as of May 24, 2021, no trials that planned to test mental health interventions with children or adolescents in COVID-19 had been registered.^4^

Strengths of our systematic review include using rigorous best-practice methods; searching 9 databases, including 2 Chinese databases; not restricting inclusion by language; and the ability to update rapidly as evidence emerges via our living systematic review approach. There are also limitations. First, we identified only 3 trials designed specifically to address COVID-19 mental health challenges. Second, the quality and plausibility of results of many trials we encountered were concerning. We were not able to verify the accuracy of what was reported in many trials and thus only described results from those trials in supplemental material. Third, we are not able to rule out the possibility that publication bias, or even censorship,^48^ may have influenced our results. Fourth, the evidence base is rapidly evolving, and main results could change, although our living systematic review format will allow rapid updating as this occurs.

In summary, we identified 3 trials of interventions that were generally well-conducted and reported, all of which were designed specifically to meet the needs of the general public or vulnerable populations in COVID-19. Together, they suggest that self-guided online interventions targeted to challenges faced in COVID-19 can effectively support mental health and that lay- or peer-delivered interventions may be an effective strategy for vulnerable populations. Additional trials are needed, particularly to address mental health challenges among children and adolescents and among diverse populations, both currently and as pandemic conditions reside.

## Supplemental Material

sj-docx-1-cpa-10.1177_07067437211070648 - Supplemental material for Effects of COVID-19 Mental Health Interventions Among Children, Adolescents, and Adults Not Quarantined or Undergoing Treatment Due to COVID-19 Infection: A Systematic Review of Randomised Controlled Trials Click here for additional data file. Supplemental material, sj-docx-1-cpa-10.1177_07067437211070648 for Effects of COVID-19 Mental Health Interventions Among Children, Adolescents, and Adults Not Quarantined or Undergoing Treatment Due to COVID-19 Infection: A Systematic Review of Randomised Controlled Trials by Olivia Bonardi, Yutong Wang, Kexin Li, Xiaowen Jiang, Ankur Krishnan, Chen He, Ying Sun, Yin Wu, Jill T. Boruff, Sarah Markham, Danielle B. Rice, Ian Thombs-Vite, Amina Tasleem, Tiffany Dal Santo, Anneke Yao, Marleine Azar, Branka Agic, Christine Fahim, Michael S. Martin, Sanjeev Sockalingam, Gustavo Turecki, Andrea Benedetti and Brett D. Thombs in The Canadian Journal of Psychiatry

## References

[1] World Health Organization. WHO Coronavirus Disease (COVID-19) Dashboard. https://covid19.who.int/. [accessed 26 October 2021].

[2] World Health Organization. Impact of COVID-19 on people's livelihoods, their health and our food systems: joint statement by ILO, FAO, IFAD and WHO. https://www.who.int/news/item/13-10-2020-impact-of-covid-19-on-people%27s-livelihoods-their-health-and-our-food-systems. [accessed 26 October 2021].

[3] Thombs BD Bonardi O Rice DB , et al. Curating evidence on mental health during COVID-19: a living systematic review. J Psychosom Res. 2020;133:110113. 32354463 10.1016/j.jpsychores.2020.110113 PMC7185913

[4] Living systematic review of mental health in COVID-19. https://www.depressd.ca/covid-19-mental-health. [accessed 26 October 2021].

[5] World Health Organization. Mental health considerations during COVID-19 outbreak. https://www.who.int/docs/default-source/coronaviruse/mental-health-considerations.pdf. [accessed 26 October 2021].

[6] Coronavirus (COVID-19): Better health – every mind matters. https://www.nhs.uk/every-mind-matters/. [accessed 26 October 2021].

[7] Centers for Disease Control and Prevention. Taking care of your emotional health. https://emergency.cdc.gov/coping/selfcare.asp. [accessed 26 October 2021].

[8] Public Health Agency of Canada. COVID-19 and mental health@work. Tips to take care of your mental health. https://www.canada.ca/en/government/publicservice/covid-19/covid-19-mental-health-work.html. [accessed 26 October 2021].

[9] World Health Organization. Scalable psychological interventions for people in communities affected by adversity. World Health Organization; 2017. https://apps.who.int/iris/bitstream/handle/10665/254581/WHO-MSD-MER-17.1-eng.pdf. [accessed 26 October 2021].

[10] Soklaridis S Lin E Lalani Y Rodak T Sockalingam S . Mental health interventions and supports during COVID- 19 and other medical pandemics: a rapid systematic review of the evidence. Gen Hosp Psychiatry. 2020;66:133-146. 32858431 10.1016/j.genhosppsych.2020.08.007 PMC7442905

[11] Yang Y Sun S Hu S Tang C Zhang Y Lin H . Comparative effectiveness of multiple psychological interventions for psychological crisis in people affected by coronavirus disease 2019: a Bayesian network meta-analysis. Front Psychol. 2021;12:577187. 33692715 10.3389/fpsyg.2021.577187 PMC7937808

[12] Drissi N Ouhbi S Marques G de la Torre Díez I Ghogho M Janati Idrissi MA . A systematic literature review on e-mental health solutions to assist health care workers during COVID-19. Telemed J E Health. 2021;27:594-602. doi:10.1089/tmj.2020.0287. 32970532

[13] Muller AE Hafstad EV Himmels JPW , et al. The mental health impact of the COVID-19 pandemic on healthcare workers, and interventions to help them: a rapid systematic review. Psychiatry Res. 2020;293:113441. 32898840 10.1016/j.psychres.2020.113441 PMC7462563

[14] Boldt K Coenen M Movsisyan A , et al. Interventions to ameliorate the psychosocial effects of the COVID-19 pandemic on children: a systematic review. Int J Environ Res Public Health. 2021;18(5):2361. 33670974 10.3390/ijerph18052361 PMC7967755

[15] Damiano RF Di Santi T Beach S , et al. Mental health interventions following COVID-19 and other coronavirus infections: a systematic review of current recommendations and meta-analysis of randomized controlled trials [Epub ahead of print]. Braz J Psychiatry. 10.1590/1516-4446-2020-1582 PMC8639008 33852690

[16] Elliott JH Synnot A Turner T , et al. Living systematic review: 1. Introduction – the why, what, when, and how. J Clin Epidemiol. 2017;91:23-30. 28912002 10.1016/j.jclinepi.2017.08.010

[17] Page MJ McKenzie JE Bossuyt PM , et al. The PRISMA 2020 statement: an updated guideline for reporting systematic reviews. Br Med J. 2021;372:n71. 33782057 10.1136/bmj.n71 PMC8005924

[18] World Health Organization. Rolling updates on coronavirus disease (COVID-19) 2020. https://www.who.int/emergencies/diseases/novel-coronavirus-2019/events-as-they-happen. [accessed 26 October 2021].

[19] Balshem H Helfand M Schünemann HJ , et al. GRADE Guidelines: 3. Rating the quality of evidence. J Clin Epidemiol. 2011;64:401-406. 21208779 10.1016/j.jclinepi.2010.07.015

[20] Berkman ND Lohr KN Ansari MT . Grading the strength of a body of evidence when assessing health care interventions: an EPC update. J Clin Epidemiol. 2015;68:1312-1324. 25721570 10.1016/j.jclinepi.2014.11.023

[21] Jüni P Altman DG Egger M . Systematic reviews in health care: assessing the quality of controlled clinical trials. Br Med J. 2001;323:42-46. 11440947 10.1136/bmj.323.7303.42 PMC1120670

[22] Moher D Hopewell S Schulz KF , et al. CONSORT 2010 Explanation and elaboration: updated guidelines for reporting parallel group randomized controlled trials. Br Med J. 2010;340:c869. 20332511 10.1136/bmj.c869 PMC2844943

[23] Hernán MA Robins JM . Causal inference. Boca Raton, FL: Chapman & Hall/CRC; 2018.

[24] Cuijpers P . The challenges of improving treatments for depression. JAMA. 2018;320:2529-2530. 30500053 10.1001/jama.2018.17824

[25] Reeves BC Deeks JJ Higgins JPT Shea B Tugwell P Wells GA . Chapter 24: including non-randomized studies on intervention effects. In: Higgins JPT Thomas J Chandler J Cumpston M Li T Page MJ Welch VA , editors. Cochrane handbook for systematic reviews of interventions version 6.1 (updated September 2020). Cochrane; 2020. Available from www.training.cochrane.org/handbook. [accessed 26 October 2021].

[26] Glasziou P Sanders S Hoffmann T . Waste in COVID-19 research. Br Med J. 2020;369:m1847. 32398241 10.1136/bmj.m1847

[27] Higgins JP Altman DG Gøtzsche PC , et al. The Cochrane Collaboration's Tool for assessing risk of bias in randomised trials. Br Med J. 2011;343:d5928. 22008217 10.1136/bmj.d5928 PMC3196245

[28] Hoffmann TC Glasziou PP Boutron I , et al. Better reporting of interventions: template for intervention description and replication (TIDieR) checklist and guide. Br Med J. 2014;348:g1687. 24609605 10.1136/bmj.g1687

[29] Hedges LV . Estimation of effect size from a series of independent experiments. Psychol Bull. 1982;92:490-499.

[30] Kahlon MK Aksan N Aubrey R , et al. Effect of layperson-delivered, empathy-focused program of telephone calls on loneliness, depression, and anxiety among adults during the COVID-19 pandemic: a randomized clinical trial. JAMA Psychiatry. 2021;78:616-622. 33620417 10.1001/jamapsychiatry.2021.0113 PMC7903319

[31] Thombs BD Kwakkenbos L Levis B , et al. Effects of a multi-faceted education and support programme on anxiety symptoms among people with systemic sclerosis and anxiety during COVID-19 (SPIN-CHAT): a two-arm parallel, partially nested, randomised, controlled trial. Lancet Rheumatol. 2021;3:e427-e437. 33899008 10.1016/S2665-9913(21)00060-6 PMC8051930

[32] Wahlund T Mataix-Cols D Olofsdotter Lauri K , et al. Brief online cognitive behavioural intervention for dysfunctional worry related to the COVID-19 pandemic: a randomised controlled trial. Psychother Psychosom. 2021;90:191-199. 33212440 10.1159/000512843 PMC7801992

[33] Al-Alawi M McCall RK Sultan A , et al. Efficacy of a six-week-long therapist-guided online therapy versus self-help internet-based therapy for COVID-19–induced anxiety and depression: open-label, pragmatic, randomized controlled trial. JMIR Ment Health. 2021;8:e26683. 33512323 10.2196/26683 PMC7886373

[34] Pheh K Tan H Tan C . Ultra-brief online mindfulness-based intervention effects on mental health during the coronavirus disease outbreak in Malaysia: a randomized controlled trial. Makara Hum Behav Stud Asia. 2020;24:118-128.

[35] Pizarro-Ruiz JP Ordóñez-Camblor N Del-Libano M Escolar-Llamazares MC . Influence on forgiveness, character strengths and satisfaction with life of a short mindfulness intervention via a Spanish smartphone application. Int J Environ Res Public Health. 2021;18:802. 33477831 10.3390/ijerph18020802 PMC7832842

[36] Shabahang R . Cognitive behavioural intervention for health anxiety, somatosensory amplification, and depression in coronavirus disease 2019 anxiety: an interventional study in Iran. Psychiatr Psychol Klin. 2020;20:87-93.

[37] Vukčević Marković M Bjekić J Priebe S . Effectiveness of expressive writing in the reduction of psychological distress during the COVID-19 pandemic: a randomized controlled trial. Front Psychol. 2020;11:587282. 33240180 10.3389/fpsyg.2020.587282 PMC7683413

[38] Yang L Ren Z Wang X , et al. 正念减压疗法对“新冠”肺炎疫情时期高校学生的心理状态与睡眠的影响 [Effect of mindfulness-based stress reduction on college students’ mental state and sleep during the epidemic of COVID-19] [Epub ahead of print]. China J Health Psychol.

[39] Turner EH Matthews AM Linardatos E Tell RA Rosenthal R . Selective publication of antidepressant trials and its influence on apparent efficacy. N Engl J Med. 2008;358:252-260. 18199864 10.1056/NEJMsa065779

[40] Santoft F Axelsson E Öst LG Hedman-Lagerlöf M Fust J Hedman-Lagerlöf E . Cognitive behaviour therapy for depression in primary care: systematic review and meta-analysis. Psychol Med. 2019;49:1266-1274. 30688184 10.1017/S0033291718004208

[41] Strudwick G Sockalingam S Kassam I , et al. Digital interventions to support population mental health in Canada during the COVID-19 pandemic: rapid review. JMIR Ment Health. 2021;8:e26550. 33650985 10.2196/26550 PMC7927953

[42] Fischer R Bortolini T Karl JA , et al. Rapid review and meta-meta-analysis of self-guided interventions to address anxiety, depression, and stress during COVID-19 social distancing. Front Psychol. 2020;11:563876. 33192837 10.3389/fpsyg.2020.563876 PMC7655981

[43] Karyotaki E Riper H Twisk J , et al. Efficacy of self-guided internet-based cognitive behavioral therapy in the treatment of depressive symptoms: a meta-analysis of individual participant data. JAMA Psychiatry. 2017;74:351-359. 28241179 10.1001/jamapsychiatry.2017.0044

[44] Boulton E Kneale D Stansfield C , et al. Rapid systematic review of systematic reviews: what befriending, social support and low intensity psychosocial interventions, delivered remotely, are effective in reducing social isolation and loneliness among older adults? How do they work? F1000Res. 2020;9:1368.

[45] Moroz N Moroz I D’Angelo MS . Mental health services in Canada: barriers and cost-effective solutions to increase access. Healthc Manage Forum. 2020;33:282-287. 32613867 10.1177/0840470420933911

[46] World Health Organization. Mental Health. https://www.who.int/health-topics/mental-health#tab = tab_3. [accessed 26 October 2021].

[47] National Institute for Health and Care Excellence. Depression in adults: recognition and management. https://www.nice.org.uk/guidance/cg90. [accessed 26 October 2021].

[48] The Guardian. China clamping down on coronavirus research, deleted pages suggest. December 20, 2020. https://www.theguardian.com/world/2020/apr/11/china-clamping-down-on-coronavirus-research-deleted-pages-suggest. [accessed 26 October 2021].

